# 

**Published:** 1908-12

**Authors:** 


### BOOKS RECEIVED

COLORADO SOUVENIR BOOK for the International Congress on Tuberculosis. Published by the Colorado State Organization.

PRACTICAL POINTS IN ANESTHESIA. By Frederick-Emile Neef, B. S., B. L., M. D., New York City, Surgery Publishing Co., New York.

PARAFFIN IN HERNIA. By Charles G. Miller, M. D., Comprising a description of the treatment of Hernia by paraffin injections. Published by the author, 70 State street, Chicago. Prepaid, $1.00.

THE PROCTOLOGIST. A quarterly journal devoted exclusively to rectal diseases, edited by Rollin H. Barnes, M. D., St. Louis. This volume contains the Transactions of the American Protologic Society, 1908.

A HANDBOOK OF SUGGESTIVE THERAPEUTICS.—Applied Hypnotism, Psychic Science. By Henry S. Monro, Americus, Georgia. Second Edition. C. V. Mosby Medical Book and Publishing Co., St. Louis, Mo.

LECTURES ON THE PRINCIPLES OF SURGERY. By Stuart McGuire, M. D., Professor of Principles of Surgery and Clinical Surgery, University College of Medicine, Richmond, Va. Published by Southern Medical Publishing Co., Baltimore, Md.

A TEXT BOOK OF HUMAN PHYSIOLOGY, THEORETICAL AND PRACTICAL. By George V. N. Dearborn, A. M. (Harvard), Ph. D., M. D., (Col.) Professor of Physiology in the Medical and Dental Schools of Tufts College, Boston, Professor of the Relations of the Body and Mind in the Sargent School for Physical Education in Cambridge, etc.; Illustrated with 300 Engravings and 9 plates. Lea & Febiger, Philadelphia.

DISEASES OF THE SKIN. By A. H. Ohmann-Dumesnil, A. M., M. E., M. D., Ph. D., etc.; Formerly Professor of Dermotology and Syphilology in the St. Louis College for Medical Practitioners; the St. Louis College of Physicians and Surgeons, etc. Third edition thoroughly revised and enlarged, 140 original illustrations. C. V. Mosby Medical Book and Publishing Co., St. Louis, Mo.

---

DISEASES OF THE SKIN AND THE ERUPTIVE FEVERS. By Jay Frank Schamberg, M. D., Professor of Dermatology and Infectious Eruptive Diseases in the Philadelphia Polyclinic and College for Graduates in Medicine. Octavo of 534 pages, illustrated. Philadelphia and London: W. B. Saunders Company, 1908. Cloth, $3.00 net. W. B. Saunders Company, Philadelphia and London.

---

A MANUAL OF DISEASES OF THE NOSE AND THROAT. By Cornelius Godfrey Coakley, A. M., M. D., Professor Laryngology in the Bellevue Hospital Medical College, New York City; Laryngologist to Columbus Hospital, the University and Bellvue Hospital Medical College Clinic, etc. Fourth edition, revised and enlarged, Illustrated with 126 engravings and 7 colored plates. Lea & Febiger, Philadelphia.

---

GENERAL SURGERY. A Presentation of the Scientific Principles upon which the practice of modern surgery is based. By Ehrich Lexer, M. D., Professor of Surgery, University of Konigsberg. American Edition, Edited by Arthur Dean Beaver, M. D., Professor and Head of the Department of Surgery, Rush Medical College in Affiliation with the University of Chicago. An Authorized Translation of the Second German Edition, by Dean Lewis, M. D., Assistant Professor of Surgery, Rush Medical College in Affiliation with the University of Chicago. 449 illustrations in the text, partly in color, and two colored plates. 8vo. pp. xxix., 1041. New York and London: D. Appleton and Co., 1908.

